# Evolution of response dynamics underlying bacterial chemotaxis

**DOI:** 10.1186/1471-2148-11-240

**Published:** 2011-08-16

**Authors:** Orkun S Soyer, Richard A Goldstein

**Affiliations:** 1 Systems Biology Program, College of Engineering, Computing, Mathematics and Physical Sciences, University of Exeter, Exeter, UK; 2 Mathematical Biology, National Institute for Medical Research, MRC, Mill Hill, London, UK

## Abstract

**Background:**

The ability to predict the function and structure of complex molecular mechanisms underlying cellular behaviour is one of the main aims of systems biology. To achieve it, we need to understand the evolutionary routes leading to a specific response dynamics that can underlie a given function and how biophysical and environmental factors affect which route is taken. Here, we apply such an evolutionary approach to the bacterial chemotaxis pathway, which is documented to display considerable complexity and diversity.

**Results:**

We construct evolutionarily accessible response dynamics starting from a linear response to absolute levels of attractant, to those observed in current-day *Escherichia coli*. We explicitly consider bacterial movement as a two-state process composed of non-instantaneous tumbling and swimming modes. We find that a linear response to attractant results in significant chemotaxis when sensitivity to attractant is low and when time spent tumbling is large. More importantly, such linear response is optimal in a regime where signalling has low sensitivity. As sensitivity increases, an adaptive response as seen in *Escherichia coli *becomes optimal and leads to 'perfect' chemotaxis with a low tumbling time. We find that as tumbling time decreases and sensitivity increases, there exist a parameter regime where the chemotaxis performance of the linear and adaptive responses overlap, suggesting that evolution of chemotaxis responses might provide an example for the principle of functional change in structural continuity.

**Conclusions:**

Our findings explain several results from diverse bacteria and lead to testable predictions regarding chemotaxis responses evolved in bacteria living under different biophysical constraints and with specific motility machinery. Further, they shed light on the potential evolutionary paths for the evolution of complex behaviours from simpler ones in incremental fashion.

## Background

Cellular behaviour is implemented at the molecular level through interacting proteins that form a dynamical system. This system allows the cell to process external signals so to generate an appropriate response. Towards understanding this signal-response relationship, extensive experimental and theoretical studies are undertaken in the specific molecular systems of model organisms. The resulting insights, however, might not be easily transferable to other organisms where the underlying system might display both structural and dynamical deviations from the model system. Any such diversity would be the result of evolution, whereby organisms adapt their response to the environmental conditions they experience and to the limitations of their specific biochemistry. For achieving a broad and predictive understanding of cellular behaviour, we need to characterise the selective pressures driving the evolution of the underlying molecular systems and the likely responses to those pressures. Here, we undertake such an evolutionary approach to study bacterial chemotaxis.

A detailed understanding of the chemotaxis pathway, the signal-response relationship it embeds, and the resulting chemotaxis behaviour in the model organism *Escherichia coli *have resulted from the pioneering work of several groups [1-5]. In brief, *E. coli *utilises several flagella to swim, where swimming behaviour can be approximated by straight runs separated by tumbling events that re-orient the cell. The tumbling probability is modulated by a negative adaptive response; a step increase in attractant concentration results in a transient decrease in tumbling rate. This response is implemented by a chemotaxis network comprised of receptors with methylation sites, reversible and modulatable motors, and five intermediary proteins providing signal transduction and integral feedback control [6,7]. This detailed understanding, however, does not permit a quantitative prediction of chemotaxis responses in other bacteria, which harbour structurally diverse chemotaxis networks [8]. In the closely related *Salmonella enterica *both molecular mechanisms and response dynamics are highly similar to that seen in *E. coli *[9,10]. *Bacillus subtilis *shows response dynamics that fits closely with *E.coli *[11], even though their chemotaxis network differ structurally [11,12]. In *Sinorhizobium meliloti *and *Rhodobacter sphaeroides*, two species belonging to the α-proteobacteria, chemotaxis is suggested to involve two separate pathways [13,14]. Experiments in these species show that such structural diversity translates to deviations from *E. coli *response dynamics [15-17]. In particular, *R. sphaeroides *seems to have slower adaptation to persistent stimuli [18] and gives an 'inverted' chemotaxis response (i.e. increasing attractant concentration causes an increase in tumbling frequency), when grown under aerobic conditions [19]. Such an inverted response is also observed in certain *Halobacteria *[20] and in certain mutant strains of *E. coli *that have been 'gutted' of some or most of the chemotaxis proteins [21,22]. Interestingly, these natural and mutant strains all still show the ability to chemotax.

To understand the significance of these structural and dynamic deviations, we consider here which alternative signal-response relationships could underlie chemotaxis and if there exist evolutionarily plausible, incremental paths between them. Previous studies have shown strict limits on the types of responses that could result in effective chemotaxis [23] and have demonstrated that an adaptive response dynamics provides the optimal chemotaxis performance [24,25]. These studies, however, assumed tumbling to be instantaneous. While this assumption is consistent with observations from *E. coli *[1], it is not expected that tumbling times are or have been always short in all bacteria. In particular, re-orientation depends on the motility machinery of the bacteria and could range from simply stopping, turning or reversing [17], with each mode potentially resulting in different time scales. To account for this, several studies have considered the possibility of extended tumbling times [24,26-28]. Some of these studies showed that relaxing the assumption of instantaneous tumbling results in diverse chemotaxis behaviors, including the possibility of chemotaxis in the absence of biochemical memory [27,28].

Given such wider range of possible response dynamics, the questions arise which of these are optimal under different circumstances, and how evolution could result in the emergence and transitions among the molecular mechanisms implementing different response dynamics. Here, we develop an analytical approach to model bacterial movement that relaxes the assumption of tumbling being an instantaneous event. We use this model to understand the nature and evolution of response dynamics for achieving optimal chemotaxis. In particular, we concentrate on the linear and adaptive responses to stimuli and analyse the chemotactic performance of these under a range of system parameters and tumbling times. The resulting analyses shed light on the role of observed diversity of chemotactic behaviour, and suggest a relation between environmental and biochemical constraints and expected response dynamics for optimal chemotaxis. In addition, they provide insight on potential routes in the evolution of chemotaxis as a series of incremental advances without invoking *de novo *simultaneous evolution of a multicomponent system and the attendant problems of 'irreducible complexity'.

## Results

Bacteria navigate their environment through periods of swimming, separated by tumbling (or stopping) events that result in re-orientation. To model this process, we consider a population in a one-dimensional space. This simplification to one-dimension is necessitated from the computational burden of the analyses we perform, however, we note that this modelling choice represents the more complicated two and three-dimensional spaces where the attractant concentration only varies along one dimension. At any time, the population will have three sub-populations; bacteria moving left or right and tumbling. Bacterial behaviour is characterised by the rate of entering (*α*) and exiting (*β*) the tumble state. The former is given by a basal rate *α*_0 _modulated by the response of the bacteria to the local attractant concentration, while *β *is assumed to be a constant. In nature, we expect *β *to relate to various biochemical features of bacteria including the structure of their motility machinery. For example, tumbling times can be longer (i.e. smaller *β*) in bacteria with unidirectional motors, where a "tumble effect" results from the stopping or slowing of the motor(s) [29,30]. The modulation of *α*_0 _corresponds to the signal-response relationship enabled by the underlying chemotaxis pathway. In particular, all studied chemotaxis networks to date seem to display negative adaptive responses, where tumbling frequency of the cell returns to a basal level after decreasing following a step increase in stimuli [4,5]. Chemotaxis in the absence of adaptation, however, is also predicted to be possible by theoretical and computational studies [27,28] and several experimental studies reported positive adaptive responses [19,31]. In addition to *α *and *β*, a key property for determining chemotactic behaviour would be the sensitivity of the chemotaxis pathway, which is shown to be highly enhanced in *E. coli *through both receptor clustering [32] and cooperative binding between pathway output and the motors [33]. In the model, we reduce such molecular mechanisms to a single parameter, *λ*, which controls the level of modulation of *α*_0 _by the signal (i.e. the sensitivity of the pathway).

**Figure 1:**
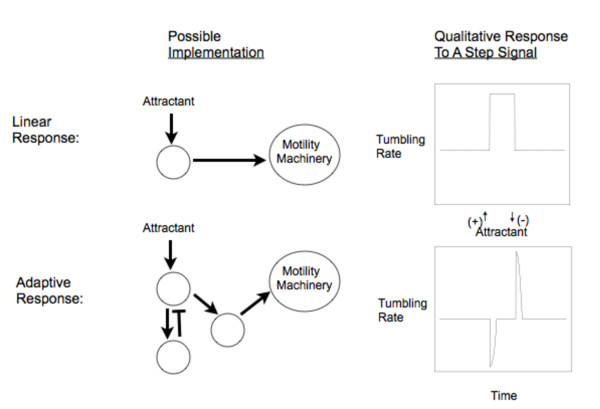
**Cartoon representation of potential molecular implementation and response to attractant for the linear and adaptive response**. See Table 1 for mathematical implementation of these dynamics.

To measure chemotactic performance, we consider a Gaussian attractant distribution moving at a fixed rate (*d*) in the one-dimensional space. While most previous studies have characterised chemotactic performance with the magnitude of the drift in a gradient [34,35], we instead considered as the chemotactic performance, *CP*, the quantity that represents the broader and evolutionarily more relevant goal of the chemotactic process; co-localisation with the attractant (but also see below). We quantified *CP *resulting from specific response dynamics and parameters *β, α*_0_, and *λ *(Table 1) over a period of time *τ*. Specifically, we consider a negative adaptive and a positive linear response with the latter representing the simplest response in terms of molecular implementation (Figure 1) still able to mediate some form of chemotaxis [28].

**Table 1:** Implementation of various response dynamics

	*α*_*L*_	*α*_*R*_
Linear response	$\alpha_{0}+\lambda \frac{A\left(x\right)}{A_{\max}}$	$\alpha_{0}+\lambda \frac{A\left(x\right)}{A_{\max}}$

Adaptive response	$\alpha_{0}+\lambda \left(v+d\right)\frac{A^{\prime }\left(x\right)}{\left(v+d\right)A_{\max}^{\prime }}$	$\alpha_{0}-\lambda \left(v-d\right)\frac{A^{\prime }\left(x\right)}{\left(v+d\right)A_{\max}^{\prime }}$

Hybrid response	$\alpha_{0}+\lambda_{Lin}\frac{A\left(x\right)}{A_{\max}}+\lambda_{Adapt}\left(v+d\right)\frac{A^{\prime }\left(x\right)}{\left(v+d\right)A_{\max}^{\prime }}$	$\alpha_{0}+\lambda_{Lin}\frac{A\left(x\right)}{A_{\max}}-\lambda_{Adapt}\left(v-d\right)\frac{A^{\prime }\left(x\right)}{\left(v+d\right)A_{\max}^{\prime }}$

*A*(*x*) and *A*'(*x*) are the local concentration of attractant and its spatial derivative. Response magnitudes are normalised by *A*_max _and *A*'_max_, the maximum values of these parameters.

**Figure 2:**
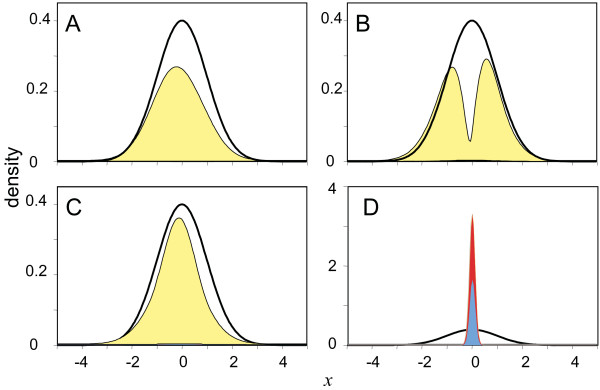
**Final distributions of bacteria after time interval *τ *= 1000 starting from a random distribution, with *d *= 0.001, for various response dynamics**. Represented are the left-swimming *L *(blue), the right-swimming *R *(red), and the tumbling population *S *(yellow). Only the population in -5 >*x *> 5 is shown. The distribution of attractant is shown with a solid black line. **A**: high sensitivity negative adaptive response, *α*_0 _= 0.01, *λ *= 100.0, *β *= 100.0, corresponding to the thick red curve in Figure 4. **B**: low sensitivity positive linear response, *α*_0 _= 0.01, *λ *= 1.7, *β *= 0.01, corresponding to the thin blue curve in Figure 4; **C**: low sensitivity negative adaptive response, *α*_0 _= 0.01, *λ *= 1.7, *β *= 0.01, corresponding to the thin orange curve in Figure 4; **D**: low sensitivity hybrid response, *α*_0 _= 0.01, *λ*_Lin _= 1.1, *λ*_Adapt _= 0.6, *β *= 0.01, corresponding to the thin green line in Figure 4; Note the change in scale in **A**. Asymmetry of the curves is due to the non-zero attractant velocity. All three low sensitivity responses, including the adaptive response, are characterised by a large fraction of tumbling bacteria co-localised with the attractant, even if the tumbling population is not large in regions far from the attractant.

**Figure 3:**
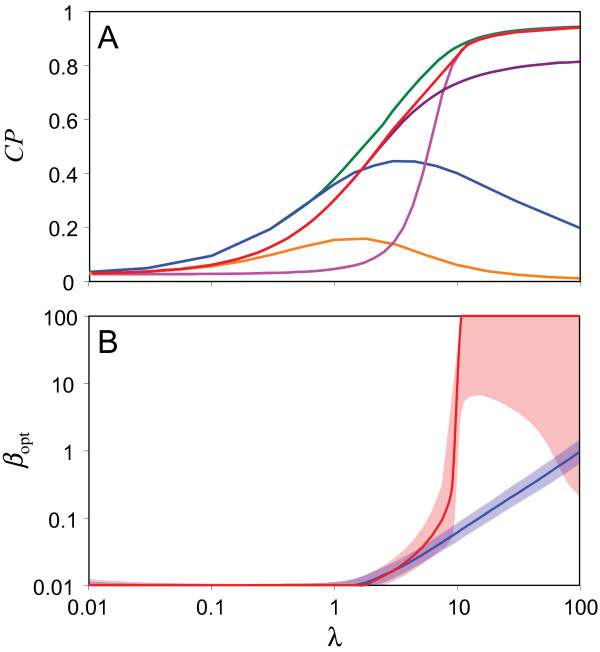
**Optimal chemotaxis performance (*CP*) and *β *for different chemotaxis strategies**. **A**. *CP *for low *β *adaptive (purple), high *β *adaptive (magenta), optimised adaptive (red), optimised inverted adaptive (orange), optimised linear (blue), and optimised hybrid (green) responses for increasing total sensitivity (*λ *or *λ*_*Lin *_+ *λ*_*Adapt*_). Performance is measured by attractant concentration *A *averaged over a finite length of time (*τ *= 1000) calculated with *d *= 0.001, normalised by maximum attractant concentration *A*_*max*_. Hybrid performance at low (high) sensitivity matches that of a linear (adaptive) response. At intermediate sensitivities a hybrid response out-performs both linear and adaptive responses. **B**. Optimal *β *value for achieving highest chemotactic performance for adaptive (red) and linear (blue) responses at different levels of sensitivity. The shaded region indicates where the chemotactic performance was within 1% of the maximum for a given *β *value. Performance is measured as in panel A.

With the negative adaptive response with high *β *(i.e. instantaneous tumbling), chemotaxis results from an effective 'gradient-climbing' with minimal time spent in tumbling, as is observed in *E. coli *[1] (Figure 2A). For this response, *CP *is negligible at low *λ *but increases to the maximum possible at high *λ *(Figure 3A, red line). For the positive linear response with low *β *(i.e. long tumbling episodes), chemotaxis results from extensive tumbling with increasing attractant (Figure 2B). *CP *resulting from a positive linear response is larger than that of the negative adaptive response at low *λ*, increases as *λ *gets larger, reaches a peak, and then decreases (Figure 3A, blue line); a low sensitivity results in bacteria simply swimming over attractant regions without detecting them, while high sensitivity results in bacteria tumbling at very low attractant concentrations never reaching regions of high attractant.

For the positive linear response, the optimal values of *α*_0 _and *β *for achieving the highest *CP *are low and mostly independent of *λ *(Figures 3B & Additional File 1, Table 2). The strategy is clear; swim unless there is high attractant, in which case tumble for a long time. The optimal value of *α*_0 _for the negative adaptive response is low and does not change with *λ *but the optimal value of *β *changes from a low value to large values in a wide range as *λ *increases (Figures 3B & Additional File 1, Table 2). Interestingly, at a sensitivity level where *CP *resulting from positive linear and negative adaptive responses are similar, we find that optimal values of *β *are comparable (close to 0.01 for both responses). This suggests that at this level of sensitivity, these two very different response types achieve chemotaxis in similar ways. Indeed, we find that at such low *β*, bacteria with an adaptive response behave much like bacteria with a linear response; much of the attractant co-localisation occurs through the creation of a large tumbling population (Figure 2C). The only difference between the two strategies is that the tumbling is caused by moving in an attractant gradient (adaptive response) rather than the attractant level *per se *(linear response).

**Table 2:** List of adjustable parameters, limits, and selected optimal values

	*α*_0_	*λ*	*λ*_Lin_	*λ_Adapt_*	*β*
Limits	0.01-100.0	0.0-100.0	0.0-100.0	0.0-100.0	0.01-100.0

*d *= 0.001, | = 1000.0					

Linear response	0.01	3.6	-	-	0.02

Adaptive response	0.01	100.0	-	-	100.0

Inverted adaptive response	0.01	1.8**			0.01

Hybrid response:					

*λ*_Lin_+ *λ_Adapt _*= 0.1	0.01	-	0.1	0.0	0.01

*λ*_Lin_+ *λ_Adapt _*= 1.0	0.01		0.76	0.24	0.01

*λ*_Lin_+ *λ_Adapt _*= 100.0*	0.01		20.0	80.0	5.2

*λ*_Lin_+ *λ_Adapt _*= 100.0*	0.01		15.0	85.0	100.0

*These solutions have nearly identical localisation with attractant. **This corresponds to a normal adaptive response with *λ *= -1.8.

**Figure 4:**
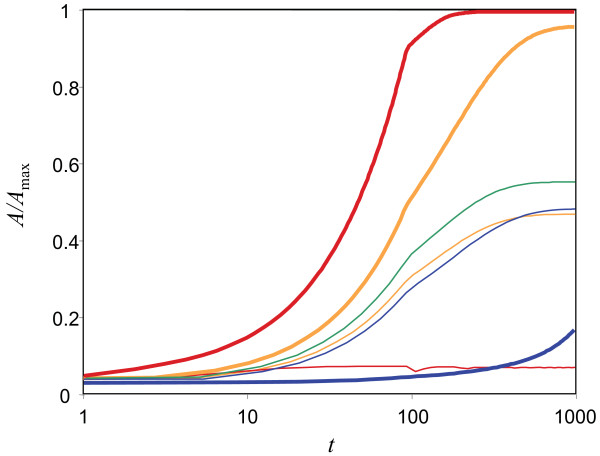
**The amount of attractant encountered with time, normalised by maximum attractant concentration, for different responses and parameter values, with *d *= 0.001: adaptive response is indicated in orange (*β *= 0.01) or red (*β *= 100.0), linear response in blue (*β *= 0.01)**. Results are presented for both low sensitivity (*λ *= 1.7, where adaptive and linear responses display similar performances, thin lines) and high sensitivity (*λ *= 100.0, thick lines). The linear response achieves very little chemotaxis with large values of *β*. The optimal hybrid mixture for *λ*_*Lin *_+ *λ*_*Adapt *_= 1.7 (*λ*_*Lin *_= 1.1, *λ*_*Adapt *_= 0.6) is shown as a thin green line. *α*_0 _= 0.01 for all curves. Resultant bacteria distributions for some of these selected responses are shown in Figure 2.

To better understand the effect of different parameter values on the chemotactic performance of the linear and adaptive performance, we also considered the time it takes for bacteria to accumulate in high attractant regions (this measure is similar to 'drift velocity' considered in other works [34,35]). We find that for the linear response, this measure is optimal at low *β *and low *λ*; increasing *λ *results in bacteria that tumble excessively at lower attractant concentrations, resulting in extremely slow dynamics (Figure 4). In contrast, a bacterium with an adaptive response and high (low) *λ *climbs an attractant gradient faster when it has high (low) *β*. This finding explains the observed shift in *β *for the adaptive response as *λ *increases. The best performance for the adaptive response is achieved with a combination of high sensitivity (large *λ*) and short tumbling time (high *β*). Additional File 2 provides a direct comparison of the performance of linear and adaptive response with select parameters.

To summarise, these results show that positive linear responses outperform negative adaptive responses in their chemotactic efficiency under low sensitivity. Further, as system sensitivity increases there is a transition from linear to adaptive responses as mediators of optimal chemotaxis. These findings are highly suggestive in an evolutionary context for two reasons. Firstly, it is highly likely that evolutionary generation of a sensing system producing a positive linear signal-response relationship is much easier compared to one producing a negative adaptive relation. In fact, the latter response type can simply be achieved through coupling of the motors to the cell metabolism as observed in several systems [36-39]. Secondly, it is plausible that, even if dedicated sensing for certain attractants was available, the level of sensitivity in ancient chemotaxis systems was low. Taken together these arguments suggest an important evolutionary role for a linear positive response as an evolutionary precursor to adaptive dynamics. If this was the case, how could molecular transitions between systems embedding the two response types have occurred?

To address this question, we considered an inverted (i.e. positive) adaptive response and a hybrid response (Table 1) as potential evolutionary paths leading from positive linear response to an adaptive response. The former response dynamics would be needed if there was a sequential progression of dynamics where adaptation comes before switching from positive to negative responses. The invention of adaptation could be mediated by addition of new proteins in the simple system shown in Figure 1 that could enable feedback on the "sensor" protein. The subsequent switching from positive to negative responses could involve only few mutations [31,40]. As an alternative to sequential progression of response dynamics from linear to adaptive, a hybrid response could result from combining the outputs of two distinct systems, one with an adaptive and one with a linear response.

**Figure 5:**
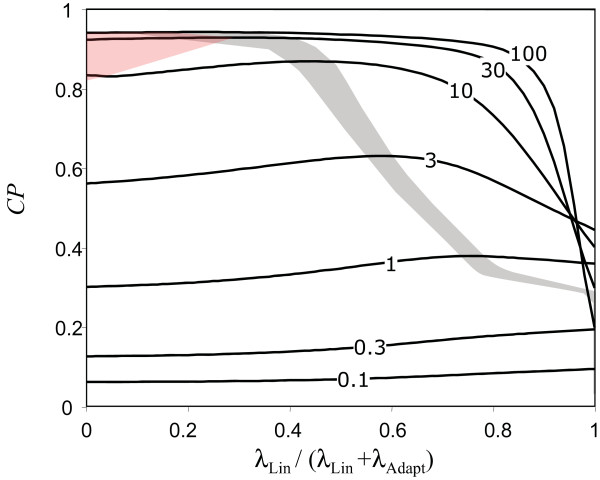
**Optimum chemotaxis performance, measured as in Figure 3A, for the hybrid response with increasing contribution from the linear response (*λ*_*Lin*_)**. The different curves correspond to different total sensitivities (*λ*_*Lin *_*+ λ*_*Adapt*_) as indicated on each curve. The shaded region indicates where the chemotactic ability was within 0.1% of the optimum for a given total sensitivity for a range of values of *λ*_*Lin*_. Note that having an adaptive response component is optimal only above a certain level of system sensitivity, with the optimal contribution of the adaptive response increasing with increasing sensitivity. The red shaded region indicates where the optimal value of *β *was approximately 100.0.

As shown in Figure 3A (orange line), we find that CP for the inverted adaptive response depends on sensitivity in a similar fashion as it does for linear response and that the maximum CP is lower than that possible with the linear response. This suggests that mutations converting a system with positive linear response to positive adaptive response would not be selected for, and therefore it is not likely that an inverted adaptive response was an intermediate step in the evolutionary trajectory (but see also *Discussion *below). For the hybrid response, we consider an overall system sensitivity that results from simple addition of the independent sensitivities of two distinct systems one with a positive linear (i.e. *λ*_Lin_) and one with negative adaptive response (i.e. *λ*_Adapt_). We then optimise both *λ*_Lin _and *λ*_Adapt _for a fixed value of *λ*_Lin _+ *λ*_Adapt_. By performing this optimisation under different total sensitivities (i.e. *λ*_Lin _+ *λ*_Adapt_), we find that *CP *resulting from an optimal hybrid response follows nicely from the *CP *resulting from a linear response at low and intermediate sensitivity and achieves maximal *CP *at high sensitivity (Figure 3A, green line). We find that under low sensitivity, the optimal hybrid strategy is to modulate basal tumbling rate only by the absolute level of the attractant (i.e. *λ*_*Lin *_> >*λ*_*Adapt*_), while at higher sensitivities, the optimal strategy is dominated by the adaptive response (Figure 5). As before, we find that as the total *λ *increases, the optimal value of *α*_0 _does not change, but the optimal value of *β *changes from a low value to large values as the optimal strategy mixture shifts. At intermediate sensitivities with lower values of *β*, the optimal strategy is dominated by the creation of a large population of bacteria tumbling where there is high attractant concentrations (Figure 2D).

## Discussion

Understanding the evolution of specific system dynamics as observed in bacteria is difficult due to limited availability of data on the dynamics of different systems and the absence of a fossil record of system dynamics from ancient bacteria. To overcome these difficulties, here we undertook an extensive mathematical characterisation of *CP *mediated by the two types of response dynamics that can enable chemotaxis; positive linear and negative adaptive response. These analyses revealed several new insights regarding the potential role of these responses in the evolution and diversity of current day chemotaxis networks.

Firstly, we find that chemotaxis systems with a low sensitivity (low *λ*) could mediate effective chemotaxis by implementing either a positive linear or a negative adaptive response. Optimisation of response parameters for high *CP *showed that while the latter response type gives the highest *CP *in such low sensitivity regime, both types of response dynamics are characterised by long tumbling times (high *λ*). These results suggest that positive linear response might be of significant importance in either ancient or current day bacteria that are limited in their ability to increase sensitivity and reduce tumbling time (e.g. due to the nature of their motility machinery). Further, we note that a positive linear mechanism is potentially the simplest response type to be implemented at the molecular level; a system without dedicated receptors but with direct coupling of the motors to the cell metabolism would suffice. Such direct coupling between chemotaxis and metabolism is observed in several bacteria [37,38].

Secondly, we find that the *CP *mediated by the positive linear response can be optimised only to a certain level with increasing *λ*. At an intermediary sensitivity, the *CP *mediated by the positive linear response starts to decrease, while that of the negative adaptive response increases. Interestingly, the *CP'*s of the two response types overlap at this range of intermediary sensitivity, suggesting that there could be an evolutionary transition between them if improving *CP *was a major selective pressure. More intriguingly, we find that at this regime of overlapping *CP*'s the optimal parameters (i.e. *α*_0 _and *β*) for both response types are comparable. This finding provides an intriguing example of functional continuity with structural change [41,42]. In other words, mutations leading to changes in the structure and response dynamics of a chemotaxis system with a linear response could be coupled to a functional continuity at the phenotypic level (i.e. *CP*).

While our analysis only considers the end effects of such mutations, it provides suggestions about likely evolutionary routes at the molecular level. In particular, we find that sequential evolution from a positive linear to negative adaptive response in a single system (e.g. through introduction of methylation sites in a receptor and additional receptor-regulated methylation proteins) is unlikely due to lower *CP *of the intermediary positive adaptive response (i.e. inverted adaptive response). We note, however, that such an evolutionary route cannot be ruled out as inverted adaptive responses are commonly observed in different chemotaxis systems and seem to be easily accessible via mutations [31,40]. Furthermore, a recent genomic analysis of chemotaxis systems found receptor proteins that combine methylation and sensing functions (i.e. do not have dedicated methyltransferase and methylesterase proteins) and kinase proteins that have an intermediary domain structure between kinases common in other signalling functions and those specific to chemotaxis [8]. It is possible that such intermediary systems had response dynamics as described here, e.g. adaptive dynamics with long tumbling times The alternative molecular route to achieving a transition between linear and adaptive responses would be to combine the output of two distinct systems with such responses. Utilisation of multiple chemotaxis networks is seen in *Rhodobacter sphaeroides *[43] and is suggested also in a large number of motile bacteria [8,44]. It is tempting to speculate that the origin of these multiple network systems relate to the evolution of chemotaxis as suggested by our analysis.

Finally, we find that at the high sensitivity regime the highest *CP *is dominantly achieved by the negative adaptive response. We note that sensitivity itself could be under selection as it provide both higher *CP *and the ability to respond to a wide range of attractant gradients (note that differing attractant levels would be equivalent to changing the sensitivity *λ *in our model). At such high sensitivity regime, the optimal parameters for the negative adaptive response leading to highest *CP *indicate a high *β*. This corresponds to instantaneous tumbling and the gradient climbing behaviour seen in *E. coli *[1,24,25]. We find, however, that at this sensitivity regime, high *β *is optimal but not essential and *CP *for the adaptive negative response can be 99% of the maximum possible even with *β *as low as 1 (Figure 3B). Such insensitivity of *CP *to the exact value of *β *would provide robustness to stochastic variations in tumbling rates, as for example observed in *E. coli *[15,45].

## Conclusions

In summary, our findings draw a crucial role for a linear positive response in the evolution of chemotaxis, both as an evolutionary precursor to adaptive response and as an optimal response under low sensitivity/long tumbling regime. The latter finding can be tested experimentally through implementation of linear chemotaxis responses using synthetic biology approaches or through experimental evolution of chemotaxis under specific conditions. The former finding suggests potential evolutionary explanations to the observed "inverted responses" and multiple network systems in certain bacteria. In particular, the latter could be the remnants of an evolutionary path where chemotaxis was achieved by combining the output of multiple networks. Even though subsequent evolution is expected to drive these systems into one with a dominant specialised chemotaxis network, there might be a selective advantage in maintaining both a highly optimised chemotactic response as well as a more general response achieved through coupling of the tumbling behaviour to metabolite concentrations. The latter mechanism could provide appropriate responses to positive and negative factors in the environment to which the bacterium has not previously been exposed, detected through their effect on the cell metabolism [46].

To predict biological networks in different organisms, we need to study how specific environmental and biochemical conditions alter the outcome of evolutionary processes shaping these networks [47]. The analysis presented here provides an approximate approach towards such understanding by considering the phenotype (in this case *CP*) of specific network dynamics under different parameter regimes (corresponding to different environmental or biochemical conditions/limitations). This evolutionary approach is generic and could be applicable in other networks where information on network structure is sparse. In the presented study of chemotaxis, it provided important insights towards understanding the significance of certain network features observed in current day bacteria, and provided an incremental evolutionary route from simple-to-implement dynamics to more complicated ones. The latter finding extends the demonstration of how simple evolutionary processes can result in complex structures [48] to the domain of system dynamics.

## Methods

In order to evaluate the chemotaxis performance resulting from different strategies, we develop an analytical model for bacterial movement as done previously [23-27]. Our approach differs from these previous works in that we explicitly consider time spent tumbling, analyse time-dependent attractant concentrations, and implement both adaptive and non-adaptive response dynamics.

We consider chemotaxis in a one-dimensional space. We imagine three populations, *R *(moving right), *L *(moving left), and *S *(tumbling). The rate at which the *R *population starts to tumble is *α*_*R*_, the rate at which the *L *population starts to tumble is *α*_*L*_, the rate at which the *S *population stops tumbling and resumes swimming is *β*; following the end of a tumble the tumbling bacteria is equally-likely to start moving in either direction. Bacteria have swimming speed *v *(= 1), and the chemoattractant distribution is moving to the right at velocity *d*. The attractant is distributed in a Gaussian distribution (variance = 1.0; qualitative results are robust to this parameter) in a space that extends from -50.0 to 50.0 with periodic boundary conditions.

As a simplification, we work in the reference frame of the attractant distribution, so that the attractant can be considered fixed while the medium moves at velocity -*d*. The bacteria swim at speed *v *relative to the medium, and tumble at rest relative to the medium, so our moving frame of reference adds an extra component of motion of velocity -*d*, that is, to the left at velocity magnitude *d*. The *R *population moves at velocity *v *- *d*, the *L *population moves at velocity -*v *- *d*, while the *S *population moves at velocity -*d*. We assume that *α*_*R *_and *α*_*L *_can depend on the local attractant distribution, while *β, v *and *d *are constant. There is no explicit time dependence in the various constants. The various conservation equations are then;

(1) $$\begin{array}{c} \frac{\partial L}{\partial t}=\left(\nu +d\right)\frac{\partial L}{\partial x}-\alpha_{L}L+\frac{\beta }{2}S\\ \frac{\partial R}{\partial t}=-\left(\nu -d\right)\frac{\partial R}{\partial x}-\alpha_{R}R+\frac{\beta }{2}S\\ \frac{\partial S}{\partial t}=d\frac{\partial S}{\partial x}+\alpha_{R}R+\alpha_{L}L-\beta S \end{array}$$

In all three equations, the first right-hand side reflects the difference in the number of bacteria entering and leaving any particular infinitesimal slice of space, while the other terms represent movement of bacteria between the *L, R*, and *S *states. The dependence between the local concentration of attractant (*A*(x)) and tumble rate (i.e. *α*_R _and *α*_L_) is characterised by three adjustable parameters, the basal tumbling rate *α*_0_, the signal gain *λ*, and the rate of exiting the tumbling state *β *(Table 1). The adaptive response represents a response to the temporal derivative of the attractant, which is simply given by the spatial derivative times the velocity of the bacterium relative to the attractant distribution. For the hybrid response, *λ*_Lin _was maximised for a fixed value of *λ*_Lin _+ *λ*_Adapt _so that both *λ*_Lin _and *λ*_Adapt _are positive.

We numerically integrated Equation 1 using an adaptive Runga Kutta algorithm, starting with a flat distribution of bacteria, either for a length of time *τ *or until convergence (corresponding to *τ *= ∞). The parameters characterising the chemotactic response (*α*_0_, *λ*, and *β*) were optimised to maximise the average attractant observed by the bacteria given fixed values of *v *and *d*. Table 2 gives the constraints on the parameters and optimal parameter values for selected conditions.

## List of Abbreviations

*CP*: Chemotactic performance.

## Authors' contributions

RAG and OSS designed the research project and performed data generation and analysis. RAG and OSS together wrote the manuscript. All authors have read and approved the final manuscript.

## Supplementary Material

Additional file 1 **Chemotaxis performance for different strategies**. Figure with two panels showing chemotaxis performance for different strategies and different *β*. **Panel A**: Chemotaxis performance (as defined in Figure 2) for bacteria with adaptive response, as a function of *β*, for various values of *λ *as indicated on the plot, for *d *= 0.001 and τ = 1000.0. At higher sensitivities optimum value of *β *shifts to a wider range of higher values. **Panel B**: Chemotaxis performance for bacteria with linear response, as a function of *β*, for various values of *λ *as indicated on the plot, for *d *= 0.001 and τ = 1000.0. Click here for file

Additional file 2 **Alternative chemotaxis performance analyses**. Figure with two panels showing alternative analyses of chemotaxis performance for different strategies. **Panel A**: Chemotaxis performance (as defined in Figure 2) for bacteria with linear (blue) and adaptive (red) responses, as a function of attractant drift velocity *d*, for infinite *τ*. Response parameters are optimized for each value of *d*. **Panel B**: Average attractant concentration, normalized by maximum attractant concentration, experienced by bacteria with linear (blue) and adaptive (red) responses, as a function of *τ* for *d *= 0. Response parameters are optimized for each value of *τ*. Both plots indicate that linear responses can work effectively for longer *τ* and smaller *d*, but that the adaptive response provides superior performance, can find the attractant faster, and is much less sensitive to attractant motion. Click here for file
